# SMILE Modeling Working Group: Modeling and Analysis of X-ray and Ultraviolet Images of Solar Wind – Earth Interactions

**DOI:** 10.1007/s11214-025-01172-8

**Published:** 2025-05-20

**Authors:** Hyunju K. Connor, Tianran Sun, Andrey Samsonov, Jun Liang, Andrew Read, Dalin Li, Gonzalo Cucho-Padin, Jaewoong Jung, Brenden Bickner, C. Philippe Escoubet, Colin Forsyth, Steven Sembay, David Sibeck, Emma Spanswick, Dmytro Sydorenko, Chi Wang

**Affiliations:** 1 NASA Goddard Space Flight Center, Greenbelt, MD USA; 2 National Space Science Center, Chinese Academy of Sciences, Beijing, China; 3 Mullard Space Science Laboratory, University College London, Dorking, UK; 4 University of Calgary, Calgary, Canada; 5 Leicester University, Leicester, UK; 6 Catholic University of America, Washington, D.C. USA; 7 University of Rhode Island, Kingston, RI USA; 8 European Space Research and Technology Centre, European Space Agency, Noordwijk, the Netherlands; 9 University of Alberta, Edmonton, Alberta Canada

**Keywords:** Solar wind, Magnetosphere, Ionosphere, Aurora

## Abstract

The Solar wind Magnetosphere Ionosphere Link Explorer (SMILE) is a joint European and Chinese spacecraft scheduled to launch in 2025 into a highly elliptical polar orbit. It will carry four instruments: the Soft X-ray Imager (SXI), the UltraViolet Imager (UVI), the Light Ion Analyzer (LIA), and the MAGnetometer (MAG). SMILE will image the dayside magnetosheath and cusps in soft X-ray, as well as the northern auroral oval in ultraviolet, for ∼41 continuous hours per orbit while simultaneously measuring plasma and magnetic field along its path. SMILE aims to advance our understanding of global solar wind – magnetosphere – ionosphere interactions. The Modeling Working Group (MWG), established in 2018, has fostered various modeling studies to ensure the successful scientific outcome of the SMILE mission. This paper overviews several MWG activities related to the SMILE SXI and UVI instruments. Firstly, we introduce the simulation of soft X-ray images of the Earth’s dayside magnetosphere, the SMILE orbit, and the SXI target visibilities. Secondly, we discuss multiple techniques developed for soft X-ray image analysis and the SXI’s capability to capture multi-scale interactions between the solar wind and Earth’s magnetosphere. Thirdly, we focus on the role of exospheric hydrogen density in determining near-Earth soft X-ray emissions, introducing several studies that estimate the exospheric density near the subsolar magnetopause location and its variability during geomagnetic storms. Finally, we present the modeling efforts for simulating the UVI instrument performance and the kinetic transport of suprathermal electrons and their impact on UV emissions.

## Introduction

Solar wind Magnetosphere Ionosphere Link Explorer (SMILE), a joint European–Chinese mission, will observe solar wind – Earth interaction with four instruments on board: MAGnetometer (MAG), Light Ion Analyzer (LIA), Soft X-ray Imager (SXI), and UltraViolet imager (UVI) (Branduardi-Raymont et al. 2018; Wang et al. 2025, this collection). SMILE will be launched near the end of 2025 into a highly elliptical orbit with a 70° inclination, ∼50.5 hour orbit duration, a geocentric apogee of ∼19.8 Earth radii ($R_{E}$), and a geocentric perigee of ∼2.0 $R_{E}$. SMILE will take the X-ray images of the dayside magnetosheath and cusps, and the UV images of auroral precipitation over the northern hemisphere, for ∼41 continuous hours per orbit, while taking in-situ measurements of magnetic fields and plasmas.

The SMILE Modeling Working Group (MWG) was established in 2018 to support soft X-ray image analysis of the solar wind – Earth interaction. Although soft X-ray imaging was a well-known technique in astrophysics, it was a new technique to the heliophysics community that requires comprehensive modeling analysis to assess the full capability of SXI. The initial MWG goals were to 1) conduct magnetohydrodynamics (MHD) simulations of expected soft X-ray images and examine the SXI science requirements, 2) develop various techniques that trace dayside magnetospheric boundaries — namely, magnetopause, bow shock, and cusps — from 2D SXI images, 3) investigate other science topics that can be addressed by SXI, such as kinetic features of the dayside magnetospheric systems (e.g., quasi-parallel shocks, hot flow anomalies, magnetosheath high-speed jets) and magnetosphere-ionosphere coupling studies utilizing both SXI and UVI imagers on SMILE. As the MWG work progressed, the team expanded their efforts to hybrid modeling and test-particle simulations of soft X-ray images, exospheric neutral density, UVI image support, etc., supporting the SMILE mission in multiple directions.

The MWG co-led by Hyunju Connor, Tianran Sun, and Andrey Samsonov organizes an in-person meeting twice per year and a virtual meeting for the remaining months to share recent progress on modeling and data analysis related to SMILE science. As of June 2024, a total of 45 MWG meetings (both in-person and virtual) had been held. A special issue on “Modeling and Data Analysis Methods for the SMILE missions” published in the Earth and Planetary Physics (EPP) journal hosted 22 peer-reviewed papers emerging from the MWG activities (Sun et al. 2024).

This paper overviews several MWG activities. Section 2 discusses the soft X-ray image simulations, the SMILE orbit, and the SXI target visibility. Section 3 introduces various image analysis techniques for SXI and discusses SXI’s capability to capture multi-scale features of solar wind – Earth interaction. Section 4 covers terrestrial exosphere density and its impact on soft X-ray emissions. Section 5 discusses the UVI performance simulation and the impact of suprathermal electrons on UV emissions. Finally, Sect. 6 summarizes this paper.

## SMILE Soft X-ray Imager (SXI)

### Modeling Soft X-ray Images

Soft X-rays in the near-Earth plasma result from the interaction between heavy solar wind ions and geocorona neutrals due to the Solar Wind Charge Exchange (SWCX) (e.g., Cravens et al. 2001; Robertson et al. 2006; Koutroumpa et al. 2009a; Carter et al. 2010; Branduardi-Raymont et al. 2012; Kuntz et al. 2015; Whittaker et al. 2016; Sibeck et al. 2018). A key example of this reaction is the interaction between the oxygen ion and neutral hydrogen:

$$ \text{O}^{7+} + \text{H} \rightarrow \text{O}^{6+} + \text{H}^{+} $$

The ion O^6+^ remains in a highly excited energy state until a radiative cascade starts that launches a photon with energy near 570 eV (Sibeck et al. 2018 and references therein). Since the solar wind contains many other different species of heavy ions in a high charge state (e.g., O^6+^, Fe^9+^, Mg^9+^, Si^9+^, C^6+^, and O^8+^), the SWCX spectrum covers the Extreme UltraViolet (EUV) and soft X-ray ranges (Koutroumpa et al. 2009b; Sibeck et al. 2018; Zhang et al. 2023a).

SWCX occurs in the near-Earth regions where solar wind ions can penetrate. Since the magnetopause is impenetrable for most ions besides extremely energetic ones, the main sources of SWCX emission are the magnetosheath and cusps. The volume emission rate ($P_{X}$) is found to be proportional to the plasma density in the solar wind and magnetosheath ($N_{SW}$), the exospheric neutral density ($N_{H}$), and the relative velocity ($V_{REL}$):

1 $$ P_{X} = \alpha N_{SW} N_{H} V_{REL}. $$

Here $\alpha $ is the interaction efficiency factor estimated to be about $10^{-15}\text{ eV}\text{ cm}^{2}$ (Cravens 2000; Pepino et al. 2004; Sun et al. 2015; Koutroumpa 2024). The exospheric neutral density $N_{H}$ falls off with $R^{- 3}$ where $R$ is a geocentric distance (Cravens et al. 2001). The neutral density at the radial distance of 10 $R_{E}$, i.e., near the subsolar magnetopause for typical solar wind conditions, is assumed to be 25 cm^−3^ (see Sect. 4.1). The relative velocity $V_{REL}$ depends on the flow velocity and thermal velocity.

Assuming that $\alpha $ is constant, we simplify the simulation of X-ray emissivity. This coefficient is linearly proportional to the heavy ion flux relative to the solar wind proton flux and has different values for low or high-speed solar wind (Schwadron and Cravens 2000) and different solar activities (Zhang et al. 2022). However, the time scale of changes in $\alpha $ is larger than the time scale of changes in the solar wind density and the relative velocity, therefore slowly varying $\alpha $ does not influence the methods of finding magnetopause position which we discuss below.

To obtain parameters required for soft X-ray modeling—such as plasma density and relative velocity near the dayside magnetosphere—the SMILE modeling working group has been utilizing a variety of global MHD models, including, but not limited to, the Open Global Circulation Geospace Model (OpenGGCM; Raeder et al. 2001, 2008), the Parabolic Piecewise Method with Lagrangian Remap (PPMLR) model (Hu et al. 2007), the Block-Adaptive Tree Solar wind Roe-type Upwind Scheme (BATS-R-US) model (Powell et al. 1999; Tóth et al. 2005, 2012), and the Lyon-Fedder-Mobarry (LFM) model (Lyon et al. 2004). These models solve ideal or resistive MHD equations with solar wind and IMF input using various numerical grids in different coordinate systems. For example, they use non-uniform Cartesian grids in the Geocentric Solar Ecliptic (GSE) coordinate system (OpenGGCM); uniform Cartesian grids in the Geocentric Solar Magnetospheric (GSM) coordinate system (PPMLR);, block-adaptive Cartesian grids in GSM (BATS-R-US); and distorted spherical grids in the $(r,\theta )$ body-fitted coordinate system (LFM). Their simulation domains are large enough to cover the entire geospace systems—from bow shock to the magnetotail—with the inner magnetospheric boundary near 2∼3$R_{E}$, where the coupling with the ionosphere takes place.

**Fig. 1:**
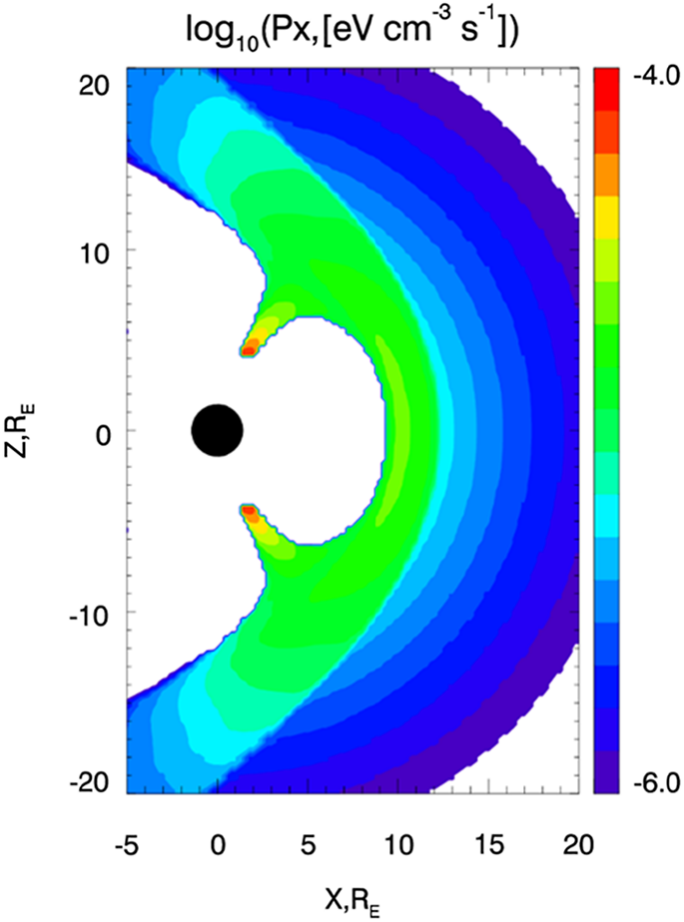
X-ray emissivity in the noon-meridional plane calculated with magnetospheric masking (adapted from Samsonov et al. 2022a)

Comparison of MHD simulations with observations shows that MHD models predict magnetic field and plasma parameters in the magnetosheath reasonably well (e.g., Crooker et al. 1985; Farrugia et al. 1998; Stahara 2002; Wang et al. 2003; Samsonov and Hubert 2004; Jung et al. 2024), except in the region downstream from the quasi-parallel bow shock where kinetic structures originated in the foreshock may play a significant role (e.g., Lin and Wang 2005; Omidi et al. 2010; Palmroth et al. 2015; Samsonov et al. 2017). Using MHD simulations, we find the density and velocity in the region around the magnetosphere for different solar wind conditions. MHD models usually predict a relatively high density in the outer magnetosphere and there are few if any high charge states ions in this region. Therefore, masking methods have been applied to separate the magnetospheric domain and set the density there equal to zero (Sun et al. 2019; Samsonov et al. 2022a). Figure 1 shows $P_{X}$ calculated by Eq. (1) in a stationary case with northward IMF (Samsonov et al. 2022a). The regions of high emissivity are the magnetosheath and two cusps.

**Fig. 2:**
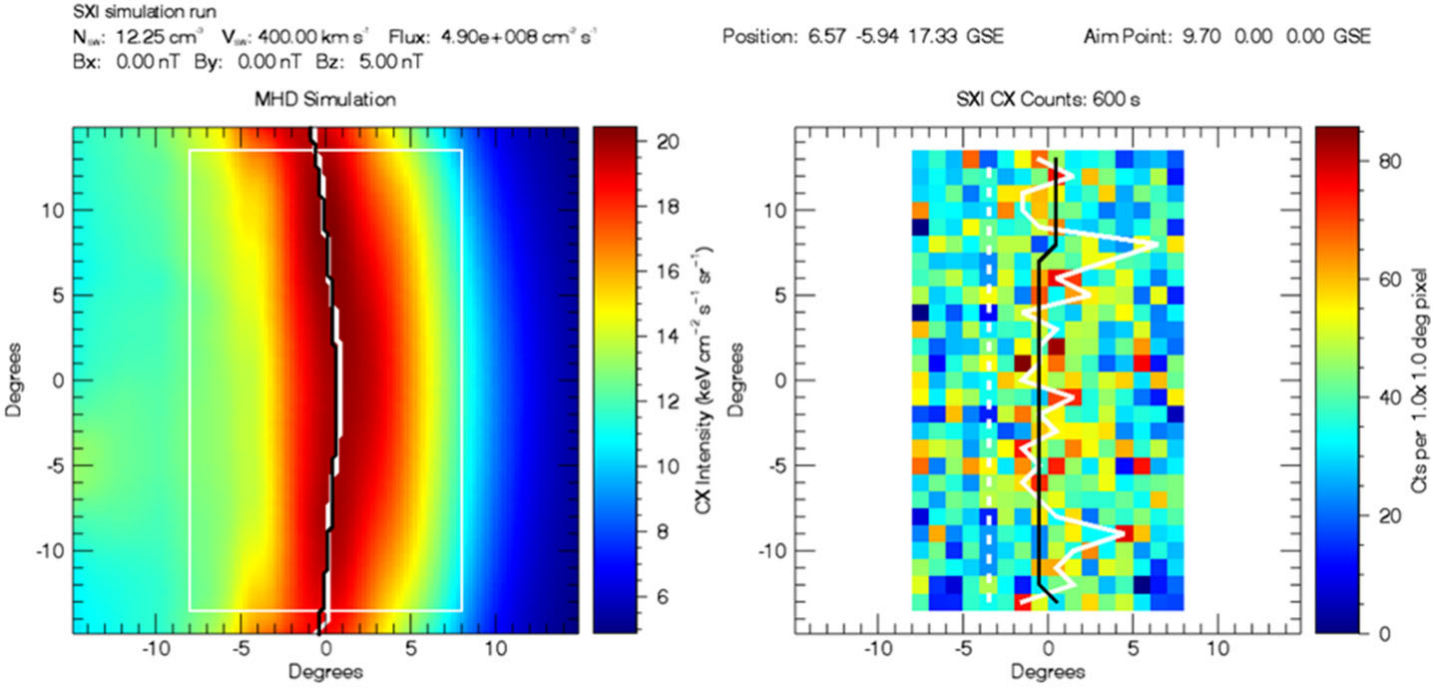
Left: integrated emissivity along the LOS for the SMILE position near the apogee $(6.57,-5.94,17.33)$ RE. Right: SXI count maps with an exposure time of 600 s. The center of the field of view is near the subsolar magnetopause. The Sun is on the right, and the Earth is on the left. White lines indicate the averaged maximum for each azimuthal (vertical) angle, and the black lines indicate the polynomial fit (see detail in Samsonov et al. 2022b)

The SMILE SXI (or any other X-ray imager) will measure the emissivity integrated along the lines-of-sight (LOS). An instrument simulation software, SXI_SIM, developed at the University of Leicester for the SMILE SXI project allows us to predict the output of the SXI instrument for a given input (Sembay et al. 2024). The primary input to the software is a three-dimensional (3D) data cube of the X-ray emissivity PX obtained from MHD simulations. SXI_SIM outputs images and spectral data. Figure 2 illustrates the SXI_SIM output for the same case shown in Fig. 1. The left panel displays the emissivity integrated along the LOS for the SMILE position near the apogee. The right panel reveals the SXI count maps, i.e. the expected instrument output that includes background noise. White and black lines indicate the positions of the maximum emissivity that brings information about the magnetopause location (see Sect. 3.1). Here, the running averages over five pixels are calculated along the horizontal axis and then used to find the location of the maximum for each azimuthal angle (along the vertical axis). These maxima are shown by the solid white lines. The black lines correspond to the polynomial fit of the white lines.

At the top of Fig. 2, the software provides information about the solar wind conditions, the spacecraft position, and the aim point (i.e. the center of the field of view). The given position $(6.57, -5.94, 17.33)$ $R_{E}$ is close to the SMILE apogee, and the aim point is nearly at the subsolar magnetopause. Such a configuration is almost perfect for observations of magnetopause motion. In this particular case when the solar wind dynamic pressure is relatively small and IMF is northward, we can only observe the magnetosheath, but not the cusps which are outside of the field of view (FOV). However, SXI can also be used to study changes in the cusp locations for different solar wind conditions and dipole tilts (Sun et al. 2021).

### SMILE Orbit and SXI Target Visibility

**Fig. 3:**
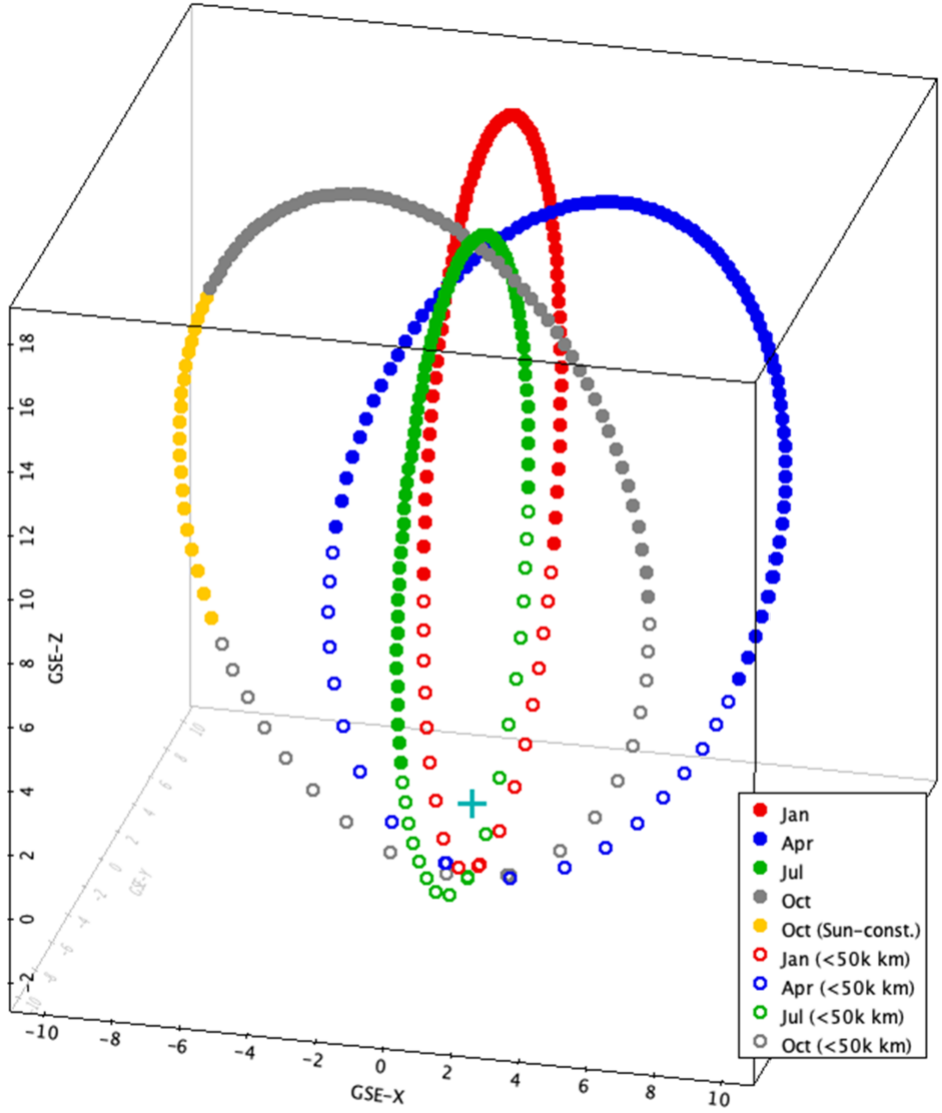
Single revolutions extracted from the SMILE orbit from the start of January (red), April (blue), July (green), and October 2026 (grey). Axis units are Earth radii. Points are separated by 30 minutes in time. The open symbols show times when the spacecraft is below $5\times 10^{4}\text{ km}$ in altitude. The yellow points in the October orbit show times when the Sun-Spacecraft-Earth angle becomes too small (see text)

SMILE has an apogee of about 20 $R_{E}$, which at times places the spacecraft in the solar wind upstream of the bow shock. Figure 3 shows four typical orbits between January and October 2026. At the start of 2026, the orbit is such that the spacecraft rises out of perigee close to the GSE-Z axis and reaches a dusk-side large positive GSE-Z apogee at positive GSE-Y (and GSE-X ∼ 0). Dayside apogee orbits at positive GSE-X (and GSE-Y ∼ 0) occur at the start of April, dawn-side apogee orbits at negative GSE-Y (and GSE-X ∼ 0) occur at the beginning of July, and night-side apogee orbits at negative GSE-X (and GSE-Y ∼ 0) occur at the beginning of October. Subsequent years behave similarly, except that the orbit apogees move closer towards the GSE-Z axis, lying almost on the GSE-Z axis by 2028.

**Fig. 4:**
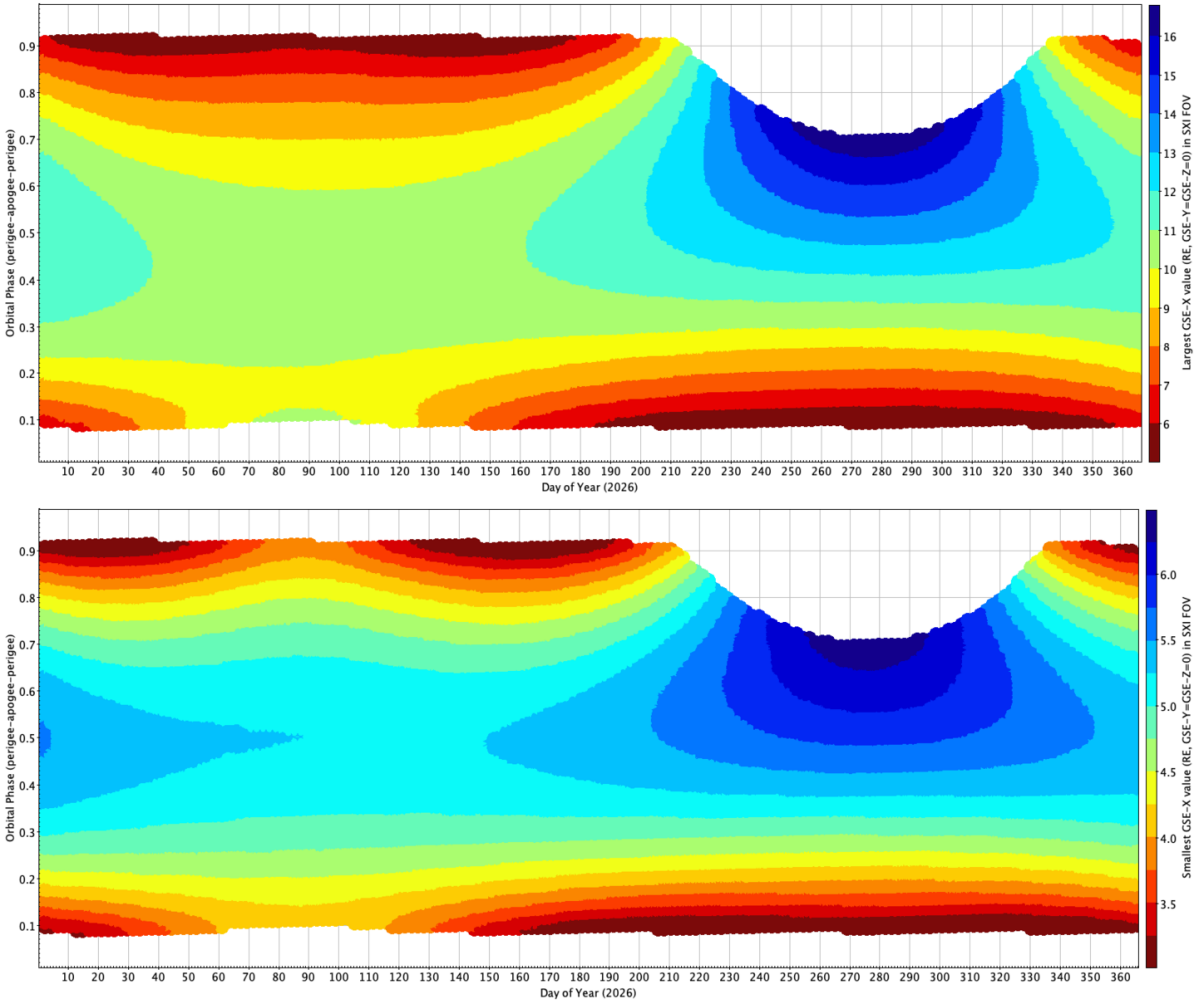
The largest and smallest values of GSE-X in R_*E*_ along the Earth-Sun line (GSE-Y = GSE-Z = 0) in the SMILE FOV as a function of days of the year 2026 and the position along the orbit

Figure 4 displays the changes in the SMILE FOV (15.5° × 26.5°) when the spacecraft moves along the orbit in 2026. The SXI pointing is such that the center of the SXI FOV lies on the GSE-X axis (the Earth-Sun line) and is positioned 20.3° from the (Sun-side) Earth limb. The short axis of the SXI FOV lies parallel to the GSE-X axis. During times when the Sun-Spacecraft-Earth angle becomes too small (approximately 6% of the time per year, during long periods in August - November 2026, evolving to shorter periods in August - November and February - May 2028), the Sun is kept at 40° from the SXI FOV center, such that the Earth drifts towards the SXI FOV, inside its 20.3° limb-limit angle, and the SXI door is closed (it might be possible to reduce the limb-limit angle a small amount during these periods to allow more SXI observation time). The SXI door is also closed when the spacecraft altitude is less than 50,000 km (approximately 17.5% [2026] to 18.8% [2028] of the year). For the rest of the time (76% [2026] to 75% [2028] of the year), SXI will make observations.

Simulations (see Read 2024) have been performed to predict when target magnetospheric (and background) objects/sources will be visible in the SXI FOV during the SMILE mission. As regards the magnetopause nose, visibility estimates can be made by considering points along the GSE-X axis. For example, the nose point [10,0,0] $R_{E}$, approximately at the magnetopause, lies within the SXI FOV for 46% of the year 2026, mainly during high- and mid-altitude times. Points along the GSE-X axis further in, at GSE-X = 9, 8, 7, 6, 5, and 4 $R_{E}$, lie within the SXI FOV for 57%, 65%, 70%, 72%, 39% and 11% of 2026, respectively. One should note that the peak in the X-ray emission usually does not appear aligned with the nose position, but instead appears to come from further out, more aligned with the tangent to the magnetopause (see Sect. 3.1). As regards the magnetospheric cusps, Tsyganenko’s (1995) dipole model was used to calculate the GSE positions of several points along three magnetic field lines (at 10, 12, and 14 MLT), from the Earth’s surface out to 7 $R_{E}$, in both the northern and southern hemispheres. The times when any parts of these structures appear in the SXI FOV, more predominantly at low- and mid-altitude times, were calculated to be 46% of 2026 for the northern cusp and 20% of 2026 for the southern cusp. All the observability percentages presented here only change by approximately one percent from 2026 to 2028.

## SMILE SXI Image Analysis

### Techniques for Tracing Magnetopause and Cusp Locations

To better understand the interactions between the solar wind and magnetosphere, it is essential to derive the positions and shapes of the magnetopause from the X-ray images observed under different solar wind conditions. The photons collected by each pixel of the SMILE SXI are integrated along a specific line of sight, creating a 2D soft X-ray image of the intended target. During this process, information along the line-of-sight direction is lost, making it a challenging task to identify the exact location of the magnetopause from X-ray image(s) and reconstruct its 3D surface. This task is one of the key activities of the SMILE MWG.

**Fig. 5:**
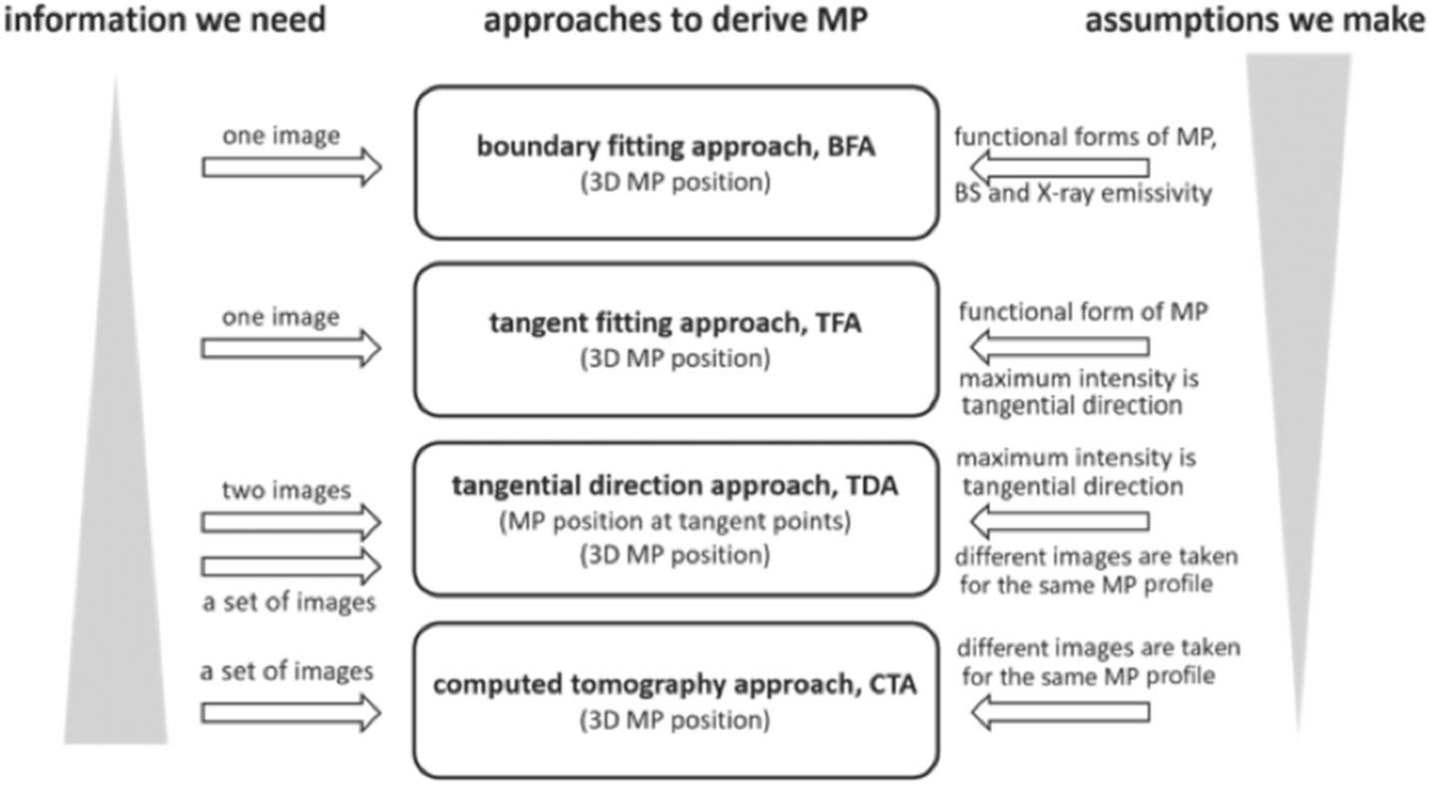
Current arsenal of magnetopause reconstruction techniques [adopted from Sun et al. (2020)]

A series of techniques are developed to trace the boundaries from the X-ray image(s), forming an arsenal of magnetopause reconstruction. The algorithms are basically a balance between the information that we have and the assumptions that we make. Under steady solar wind conditions, the magnetopause does not change significantly, enabling the imaging of a quasi-steady magnetopause profile from different vantage points on the SMILE orbit with various viewing directions. Therefore, the 3D magnetopause boundary can be reconstructed with little or no assumptions. However, when the solar wind changes in a very short time scale, the magnetopause location dynamically changes. The reconstruction of the magnetopause must be made based on a single X-ray image to capture the time-varying interaction between the solar wind and the Earth’s magnetosphere. In this case, some kinds of assumptions have to be introduced to derive the boundary position, for instance, the shape of magnetopause, distribution of the X-ray emissivity in the magnetosheath, etc. Currently, mainly four types of algorithms have been developed, as summarized in Fig. 5 (Wang and Sun 2022), i.e. the Boundary Fitting Approach (BFA), Tangential Direction Approach (TDA), Tangent Fitting Approach (TFA), and Computed Tomography Approach (CTA).

The BFA applies to situations with fast solar wind variations, enabling the reconstruction of 3D magnetopause based on a single X-ray image (Jorgensen et al. 2019a,b). The basic idea is as follows. Firstly, BFA makes assumptions on the shapes of boundaries and the spatial distributions of X-ray emissivity, giving functional forms with free variables based on empirical models or fitting results to MHD simulations. Then, BFA lets the parameters of the models float and obtains a series of X-ray images. Finally, an optimal match between the true X-ray image and the series of model simulated images is found, and the corresponding parameters define the reconstructed 3D magnetopause. 11 free parameters are proposed and the Nelder and Mead simplex method is utilized to obtain a minimum of the cost function. The results prove that the 3D structure of boundaries can be well reconstructed under a wide range of viewing geometries, backgrounds, and pixel count rates, in the situation that the model is an exact representation of the observations and the free parameters introduced are few.

The TDA derives locations of the tangential points to the magnetopause boundary using two X-ray images with both looking directions separated properly. Therefore it applies to situations with quasi-steady solar wind conditions (Collier and Connor 2018). Validated by MHD simulations as well as XMM-Newton observations (Zhang et al. 2023b), it is suggested that the local peaks of observed soft X-ray intensity correspond to line-of-sight tangents to the magnetopause. Then, one derives a mathematical formula to extract the magnetopause boundary position with information on how the peak angle changes as the spacecraft moves. With the application of TDA to a circular satellite orbit of 20 $R_{E}$ radius that processes across all local times, the magnetopause locations are well reconstructed based on X-ray images from various viewing geometries. Samsonov et al. (2022b) analyzed several X-ray images obtained from MHD simulations and reported that the magnetopause is located between the maximum of the integrated emissivity and the maximum gradient of the integrated emissivity depending on the method used.

**Fig. 6:**
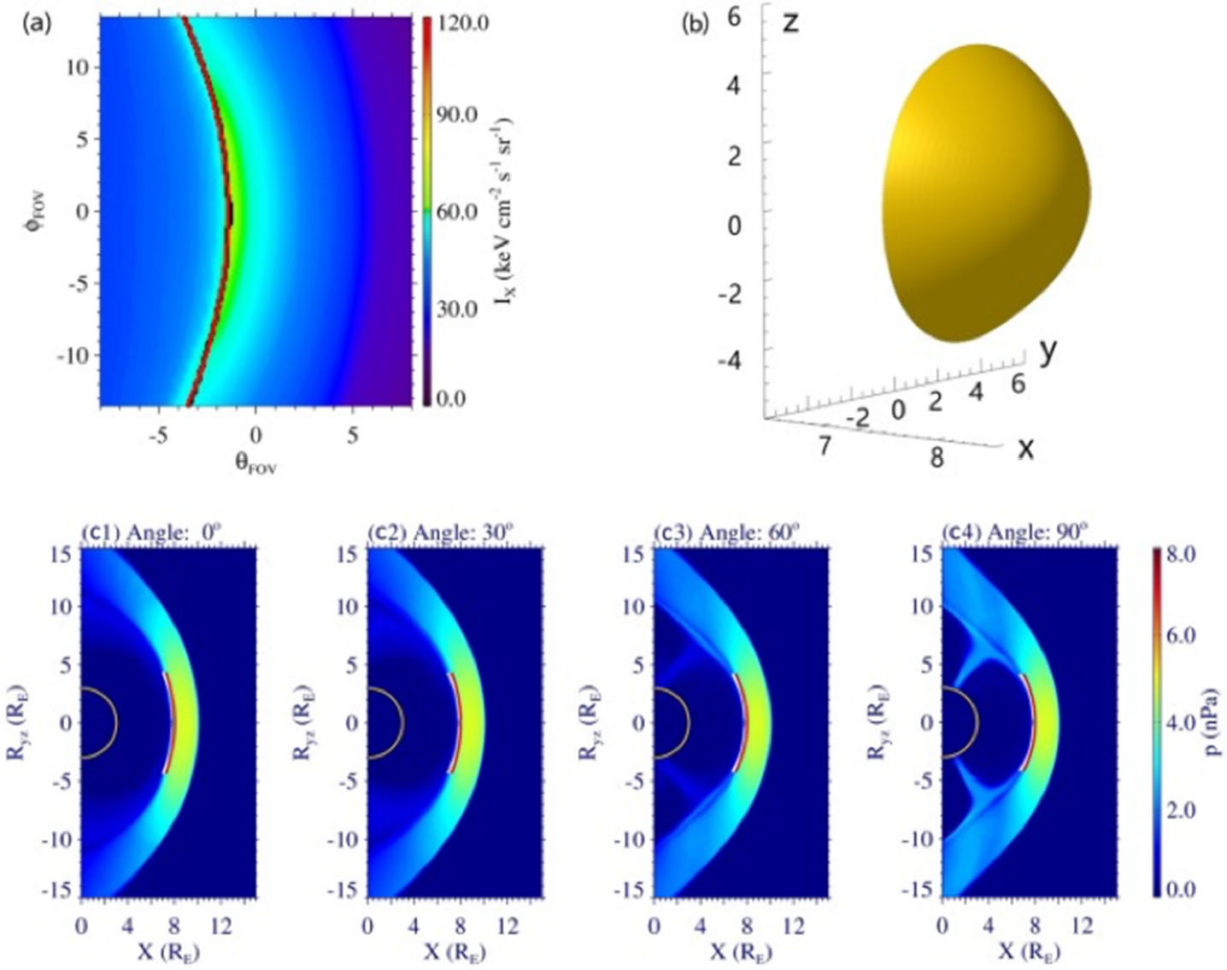
Reconstructed magnetopause using the TFA. (a) MHD simulation of the X-ray image. The black and red curves show the best-matched tangent directions. (b) Reconstructed 3D magnetopause. (c1–c4) Contours of thermal pressure on different planes with the white and red curves showing the “true” and reconstructed magnetopause, respectively [adapted from Sun et al. (2020)]

**Fig. 7:**
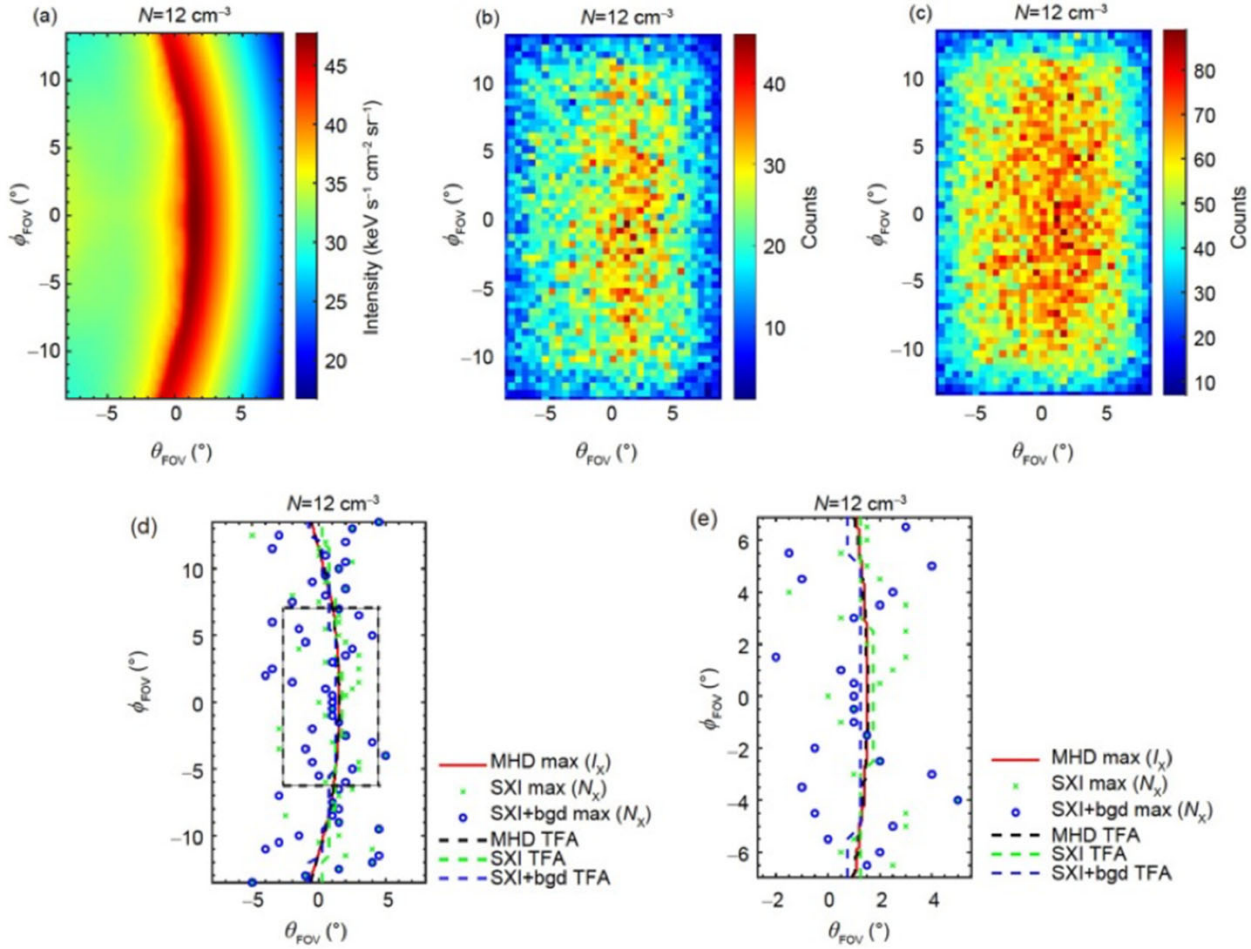
Magnetopause boundary traced from noisy X-ray images. (a) MHD simulation of the X-ray image. (b)/(c) Instrument simulations of the SMILE SXI photon count images without/with sky background. (d) Magnetopause reconstructed by the TFA. (e) A zoom-in view of (d) [adapted from Guo et al. (2022)]

The TFA combines the advantages of BFA and TDA (Sun et al. 2020). This approach makes assumptions on the shape of the magnetopause, introducing a modified Shue model (Shue et al. 1997) with 3 free parameters. Then, instead of finding an optical match of photon counts in all the pixels as did in the BFA, the TFA first calculates the tangent directions of the magnetopause from the maximum X-ray intensity as suggested by the TDA, and then finds an optical match of these tangent directions between the observed X-ray image and the series of simulated images. Therefore, by focusing on the region that carries the most information on magnetopause positions in an X-ray image, the TFA significantly reduces the number of free parameters from 11 in the BFA to 3, leading to a more stable reconstruction result that is not dependent on the accuracy of the initial guess at the beginning of the iterations (Fig. 6). Guo et al. (2022) presented the application of TFA to noisy images produced by instrument simulations as shown in Fig. 7, discussing the effects of integration time, diffusive soft X-ray background, and viewing geometry. Kim et al. (2024) also simulated the soft X-ray images using a global MHD model and added the expected Poisson noises in the images. They made an alternative assumption about the shape of magnetopause, i.e. the magnetopause near the subsolar point is nearly spherical. Utilizing the TFA, the magnetopause can be reconstructed based on a single X-ray image, and thus applicable to periods of dynamic solar wind conditions.

The CTA conducts magnetopause reconstruction using the tomographic reconstruction of X-ray emissions (Jorgensen et al. 2022; Cucho-Padin et al. 2024; Wang et al. 2023a). It analyzes the 3D distribution of X-ray emissivity based on images taken with different viewing geometries, and thus it applies to quasi-steady solar wind conditions. A denoising algorithm called total variation (TV) minimization is used together with a simple technique, i.e. the Algebraic Reconstruction Technique (ART). It is validated from MHD simulation that applying CTA to more than 30 images will return reasonable reconstruction results. In addition to its application during prolonged steady solar wind, the CTA can also be utilized after gathering a large database of SXI images and binning these images according to similar solar wind conditions. Besides, considering the possible joint observations from multiple satellites in space, such as SMILE, LEXI, LSXI (Guo et al. 2021), or GEO-X, Jorgensen et al. (2024) demonstrate that the magnetopause position can be traced by the CTA based on a single X-ray image from each spacecraft, allowing real-time reconstruction during intervals with unsteady solar wind.

It is also noted that the above approaches to trace the boundaries from soft X-ray images are also applicable to other types of images, for instance, ultraviolet (UV) images, Energetic Neutral Atom (ENA) images, etc.

The cusp boundaries are more difficult to derive compared to the magnetopause boundary, mainly due to the complicated shape of the cusp. Currently, there is no empirical model published in the literature to describe the 3D cusp boundaries, extending from right above the ionosphere to the high-latitude magnetopause. Nevertheless, based on MHD simulations, Sun et al. (2021) suggested that by finding the direction with an appreciable increase of local standard deviation (or peak intensity) in an X-ray image, the direction tangential to (or through the center of) the cusp region can be derived. Their paper initiated the development of a technique to extract the equatorward and poleward boundaries of the cusp from X-ray images, as well as the derivation of a functional form of the 3D cusp based on MHD simulations—both of which are still ongoing.

### Techniques for Processing SXI Images

After the launch of SMILE, a large number of soft X-ray images will be produced by SXI. Image pre-processing methods based on Artificial intelligence (AI) will help with the data analysis process. One possible application of AI is to filter out those images that do not contain the magnetopause structure. Wang et al. (2023b) propose an approach called UCFE-RF decoupling classification, which uses machine learning and deep learning to separate the simulated X-ray images with and without magnetopause. The method uses a self-supervised contrast feature extraction network to study the features of SXI images. With the network, the random forest classifier can distinguish whether the subsolar point at the magnetopause has been detected. Then, they designed a magnetopause filter to obtain the subsolar magnetopause images with observation positions outside the magnetosphere. In the experiments, the prediction accuracy of the classifier is up to 93%.

Finding out the position of peak emission is important to trace the boundaries in an X-ray image. However, in a photon count image considering background/instrumental noises, for instance Fig. 7 (d), the maxima are diffusely distributed, with the value of expectation around the magnetopause position. Decreasing the solar wind flux and the exposure time will lead to more diffusely distributed maxima and finally failure in tracing the magnetopause position. Zhang et al. (2023c) proposed to understand how these maxima are distributed based on deep learning, and thus build up a detection network of maximum photon counts (improved network of DeepLabV3+). The experiment results showed that the proposed network can locate the maximum photon counts efficiently, even with limited exposure time and low solar wind flux.

**Fig. 8:**
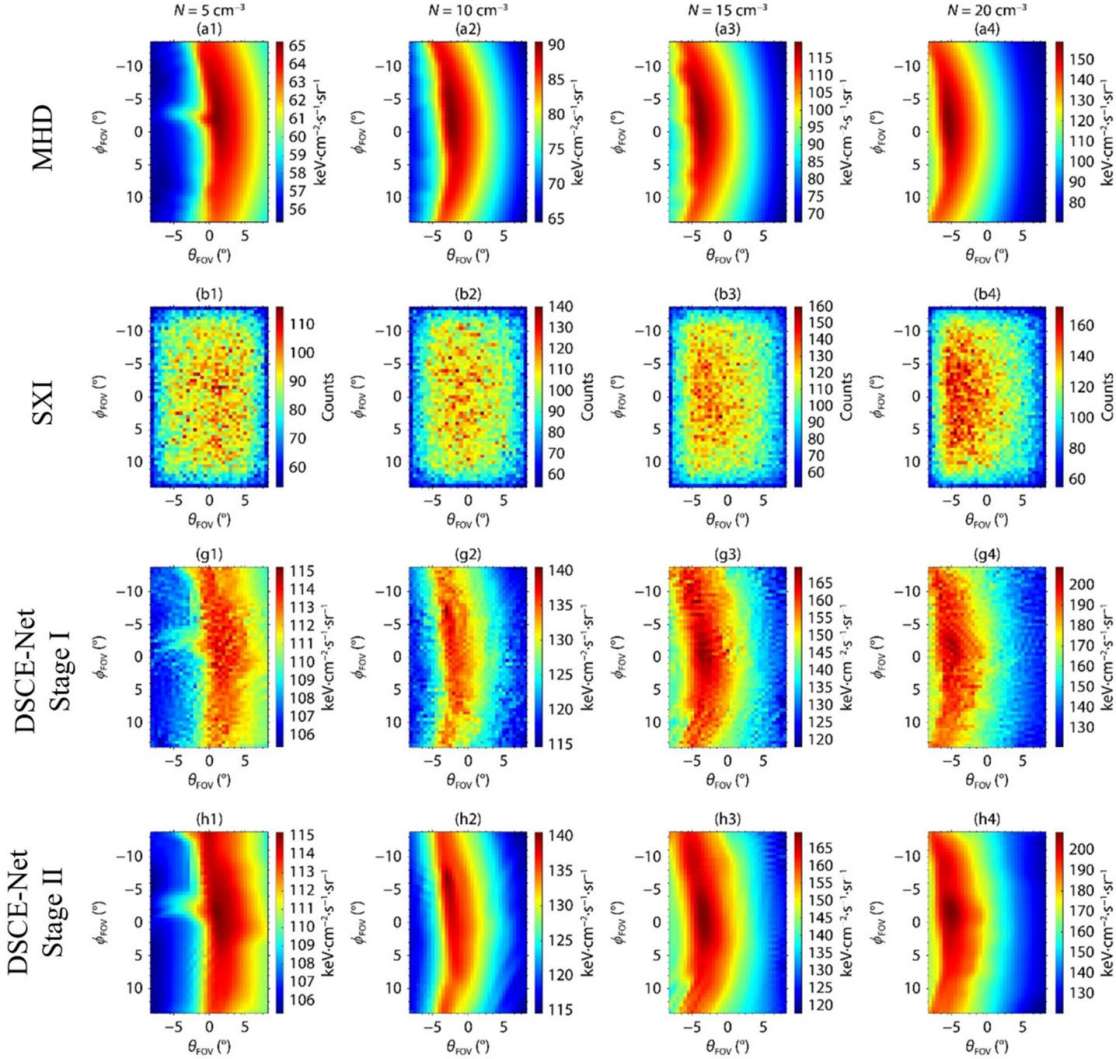
(a1)–(a4) Images of X-ray intensity predicted by MHD without diffusive soft X-ray background; (b1)–(b4) Expected SXI photon count images after adding the constant sky background; (g1)–(g4) restoration result before smoothing, as obtained by DSCE-Net; (h1)–(h4) restoration result after smoothing, as obtained by DSCE-Net. Columns from left to right correspond to solar wind number densities $N=5\text{ cm}^{-3}$, $N=10\text{ cm}^{-3}$, $N=15\text{ cm}^{-3}$, and $N=20\text{ cm}^{-3}$, respectively. Other solar wind conditions are $V_{x}=600\text{ km/s}$ and $B_{z}=0\text{ nT}$. [adapted from Wang et al. (2024b)]

An alternative way to deal with the noisy photon count image is to learn how the noise is distributed on the image and then restore a noise-free image. Wang et al. (2024b) proposed an image restoration algorithm based on deep learning. Through the training of neural networks, they established the correspondence between local noise-free images and noisy images, forming noise-clean patch pairs through the Classification - Expectation Maximization (CEM) algorithm to achieve the restoration of the simulated SXI image. Figure 8 demonstrates that the visibility of the magnetosheath region with high-X-ray intensity is notably improved in panels g1-g4 after applying the restoring method, compared to the original images in panels b1-b4 of the same figure.

To analyze those restored images that contain the structure of magnetopause, Wang et al. (2024a) proposed a model to detect the magnetopause position, named OESA-UNet. Firstly, adaptive threshold segmentation technology is used as pre-segment of the magnetosphere, providing an estimation of the region with magnetopause. Afterward, the model is designed with a type of convolutional neural network architecture that is particularly effective for image segmentation tasks. Then, new evaluation metrics are selected and designed to evaluate the segmentation results of the magnetopause target in the images. OESA-UNet captures the magnetopause with high efficiency.

### X-ray Imaging of Multiscale Interactions Between the Solar Wind and Earth

Using the SXI data processing methods explained above, the SMILE MWG simulated magnetospheric response to different solar wind conditions. These simulation studies help to understand which solar wind and magnetosheath events can be better captured by SMILE SXI, and how X-ray images will change for different spacecraft locations and aim points. For example, one of the principal SMILE science questions is “What are the fundamental modes of the dayside solar wind/magnetosphere interaction?”. It is generally accepted that magnetic reconnection on the dayside magnetopause strengthens as IMF turns southward, but whether the reconnection occurs continuously or in impulses remains controversial. Observations of temporal variations of magnetopause standoff distance in response to southward IMF turnings can answer this question.

Connor et al. (2021) simulated an artificial event with the southward turning, Samsonov et al. (2024) studied an event with the real solar wind data, and Gong et al. (2025) presented the simulations during a super storm driven by an ICME event. Connor et al. (2021) used the MHD simulations to calculate the emission rate $P_{X}$ as well as the integrated emissivity along lines of sight. Samsonov et al. (2024) applied both the MHD model and the SXI_SIM software and obtained the emissivity integrated along LOS. The simulations in both cases clearly show Earthward magnetopause motion and an increase in the magnetosheath emission rate after southward turning because, as the magnetosheath moves Earthward, more neutrals are available and increases probability of solar wind charge exchange. Samsonov et al. (2024) concluded that SMILE SXI can observe the change in the magnetopause standoff distance in such an event if the spacecraft is located in a good position (well above the subsolar point) and the SXI FOV covers the magnetopause variations.

The magnetopause standoff distance also responds to changes in the solar wind dynamic pressure and, in particular, to interplanetary shocks. Samsonov et al. (2024) noticed that the changes in the magnetopause standoff distance after an impact of interplanetary shock are usually stronger and more rapid than the changes in response to southward turning. On one hand, these changes are more easily observable because they are faster and cannot be confused with other variations, on the other hand, significant changes in the dynamic pressure may result in the magnetopause slipping out of the SXI FOV when the SXI does not observe accurately both the beginning and end magnetopause positions. Gong et al. (2025) indicates that the responses of magnetopause to a sudden impact by ICME driven shocks and southward IMF turning in the ICME are expected to be captured by SXI based on MHD simulations. Nevertheless, for short periods under extreme solar wind conditions during a super storm, the magnetopause can be severely eroded (with a standoff distance as small as 4.9 $R_{\mathrm{E}}$) so that it is pushed to or beyond the edge of the SXI FOV.

Observations of magnetopause motion contain information about processes on multiple scales. The largest scale is global magnetospheric compressions and expansions in response to significant changes in the solar wind parameters. Magnetopause MHD surface waves (Archer et al. 2024) are other large-scale phenomena with a frequency usually below 2 mHz (Hartinger et al. 2015) that give a characteristic spatial scale of about 1 $R_{E}$. The global magnetospheric waves (such as cavity mode waves) may arise from a dynamic pressure pulse or interplanetary shock (e.g. Samsonov et al. 2011). Magnetopause motion related to these waves is potentially observable by SXI.

Numerous phenomena caused by kinetic physics usually occur on smaller scales, but some may be observable by SXI. Global hybrid particle-in-cell simulations by Guo et al. (2024) and global hybrid-Vlasov simulations by Grandin et al. (2024) reproduced ion kinetic processes for southward IMF orientations. The simulations predict plasma fluctuations (Guo et al. 2024) and mirror mode waves (Grandin et al. 2024) in the magnetosheath as well as flux transfer events at the magnetopause (Grandin et al. 2024). The images of integrated emissivity presented by Guo et al. (2024) demonstrate variations in the magnetosheath, however, the authors concluded that the influence of these fluctuations on identifying magnetopause boundary in X-ray images is small under different solar wind conditions and viewing geometries. Yang et al. (2024) used global hybrid simulations to study magnetopause deformation for quasi-radial IMF conditions. They found that high-speed jets generated at the quasi-parallel bow shock can produce bright spots of X-ray emissivity in the magnetosheath and result in local magnetopause indentations. The characteristic spatial scale of the indentations (and jets) of 1 $R_{E}$, and the temporal scale of seconds or minutes (e.g., Palmroth et al. 2018; Chen et al. 2021; Yang et al. 2024) are in agreement with observations (Plaschke et al. 2016). These structures may be visible on SXI images when the magnetosphere is moderately compressed and the average emissivity rate is relatively high. Finally, Matsumoto and Miyoshi (2022) suggested from a global MHD simulation that under low plasma-$\beta $ solar wind conditions, plasma outflows from reconnection sites may be visible in soft X-ray.

## Exospheric Neutral Density and Its Role in Near-Earth Soft X-ray Emissions

Soft X-rays are emitted when a highly charged solar wind ion exchanges an electron with an exospheric hydrogen atom and reaches a rest state. As discussed in Sect. 2, exospheric hydrogen atom density ($N_{H}$) is one of the key parameters that determine the strength of soft X-ray emissions. High exospheric neutral density increases the probability of solar wind charge exchange, thus enhancing soft X-ray emissions in the dayside magnetosheath and cusps, which are the SMILE SXI target areas. For simulating the soft X-ray images, the SMILE MWG utilizes a simple exospheric neutral density model in which $N_{H}$ is inversely proportional to the cube of radial distance ($r$) (e.g., Sun et al. 2019; Connor et al. 2021; Samsonov et al. 2022b):

$$ N_{H} = N_{0} \left ( \frac{10 R_{E}}{r} \right )^{-3}. $$

In this simple model, the exospheric density at a radial distance of 10 $R_{E}$ ($N_{0}$) is set at 25 cm^−3^. This $N_{0}$ has been of particular interest to MWG because the subsolar magnetosheath, the major target region of SXI, starts near 10 $R_{E}$, where a subsolar magnetopause is located (Samsonov et al. 2019). However, previous exosphere studies have reported a wide range of $N_{0}$ from 4 to 59 cm^−3^ (e.g., Fuselier et al. 2010, 2020; Zoennchen et al. 2013, 2015; Kameda et al. 2017; Baliukin et al. 2019) as well as storm-time variability of exospheric density (e.g., Bailey and Gruntman 2013; Zoennchen et al. 2017; Cucho-Padin and Waldrop 2019; Cucho-Padin et al. 2023), which drove the MWG team’s focus on exosphere research. In this section, we review MWG research efforts on the exosphere and discuss insights on the SMILE soft X-ray imaging observations.

### Estimation of Exospheric Neutral Density at the $10R_{E}$ Subsolar Point

The terrestrial exosphere is the Earth’s outermost atmosphere. Above ∼1500 km altitude, neutral hydrogen atoms (H) become the dominant species. Current technology is not able to take in-situ measurements of tenuous, low-energy neutral hydrogen atoms above 2 $R_{E}$ radial distances (Connor et al. 2024), so most exospheric density studies utilize remote sensing of geocoronal emissions, i.e., solar Lyman-$\alpha $ photons resonantly scattered by neutral hydrogen atoms. However, there has been a severe lack of dayside geocoronal observations above 8 $R_{E}$ radial distance. Most geocoronal observatories, like Dynamic Explorer and Two-Wide angle Imaging Neutral-atom Spectrometer (TWINS), are located below 8 $R_{E}$ and thus have difficulty observing the outer dayside exosphere because the Sun is near or in the vicinity of the instrument’s field-of-view, contaminating geocoronal observations with direct sunlight.

As part of the SMILE MWG activities, two unique geocoronal observations were analyzed to extract the dayside exospheric density above 8 $R_{E}$ radial distance. Cucho-Padin et al. (2022) utilized a Lyman-Alpha Imager Camera (LAICA) image from the Proximate Object Close Flyby with Optical Navigation (PROCYON) that covered a vast region of geocorona up to ∼38 $R_{E}$ radial distance (Kameda et al. 2017). They extracted an exospheric neutral density in the 6 – 20 $R_{E}$ radial distance by using a tomographic approach, estimating $N_{0}$ at 26.51 cm^−3^. Zoennchen et al. (2022) examined geocoronal observations of the Cassini spacecraft that covered the dayside exosphere in the 3 – 15 $R_{E}$ radial distance while it passed by Earth during its journey to Saturn. They estimated an exospheric hydrogen atom density of 35 cm^−3^ at the 10 $R_{E}$ subsolar point. Additionally, they reported that exospheric density above 8 $R_{E}$ radial distance is inversely proportional to a cube of radial distance (i.e., $N_{H} \propto r^{-3}$), supporting the MWG’s exosphere density model used for the SXI image simulations.

The MWG team also employed soft X-ray observations from the X-ray Multi-Mirror Mission (XMM) to estimate $N_{0}$. Connor and Carter (2019) analyzed two events in 2001 and 2003 when the XMM line-of-sight traversed the dayside magnetosheath and observed strong near-Earth soft X-ray emissions. Using the OpenGGCM global MHD simulation, they extracted the plasma conditions along the line-of-sight and deconvolved the plasma contributions from the XMM soft X-ray observations, estimating an exospheric hydrogen density of $39.9 \pm 8.0$ and $57.6 \pm 8.0\text{ cm}^{-3}$ at 10 $R_{E}$ subsolar point. These estimates are at the higher end of exospheric density reported in the previous literature (4 – 59 cm^−3^). They interpreted this high density to be a result of the solar maximum because Zoennchen et al. (2015) reported an enhanced exospheric density in the 3 – 8 $R_{E}$ range with increasing solar activity, using 14 days of the TWINS geocoronal observations. Jung et al. (2022, 2025) adopted a similar technique to Connor and Carter (2019) to extract a solar minimum exospheric density of $36.8\pm 11.7\text{ cm}^{-3}$ using an XMM soft X-ray observation on 12 November 2008, and a solar maximum exospheric density ranging from 42.5 to 65.2 cm^−3^, with an error bar of 7.2∼18.7 cm^−3^, based on five XMM observations in 2000-2003. Considering the error bar, these XMM studies suggest minimal impact of solar activity on the subsolar exospheric neutral density at 10 $R_{E}$, with the reservation that eight events are not a sufficient sample size to derive a statistical conclusion.

**Fig. 9:**
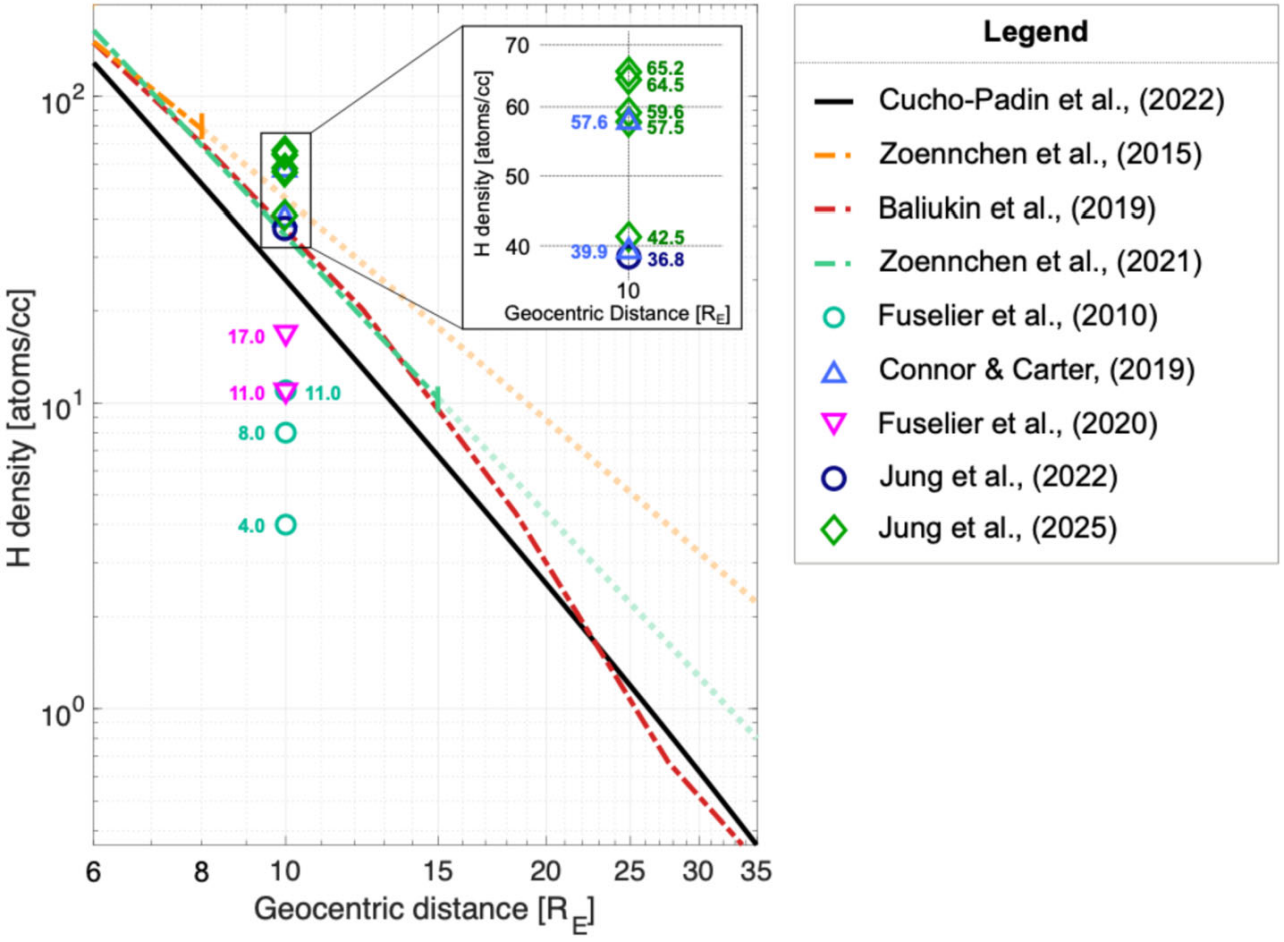
Exospheric hydrogen atom density along the dayside Sun-Earth line reported in previous literature [adapted from Cucho-Padin et al. 2022]

Figure 9 compares the exospheric neutral densities along the dayside Sun-Earth line in previous literature. The MWG studies (Connor and Carter 2019; Zoennchen et al. 2022; Jung et al. 2022, 2025; Cucho-Padin et al. 2022) showed that $N_{0}$ is comparable to or higher than 25 cm^−3^, the value adopted by MWG for the SMILE soft X-ray simulations. Thus, 25 cm^−3^ is a good conservative value for simulating soft X-ray images and developing image analysis techniques. However, there is still a concern about lower exospheric neutral density because Fuselier et al. (2010, 2020) estimated $N_{0}$ at 4 – 17 cm^−3^ by analyzing 5 Interstellar Boundary Explorer (IBEX) events when IBEX observed Energetic Neutral Atoms (ENA) emitted from the dayside magnetosheath by the charge exchange between magnetosheath ions and exospheric hydrogen atoms. This discrepancy may be reduced by adopting a more sophisticated density extraction technique, as speculated by Sibeck et al. (2021). For example, Fuselier et al. (2010) assumed time-independent, homogeneous magnetosheath plasmas along a simplified IBEX line-of-sight for their density extraction techniques. The global MHD simulation of magnetosheath plasma and the consideration of time-varying lines-of-sight of the IBEX ENA instrument may change the $N_{0} $ estimates and are currently under the MWG’s radar.

### Storm-Time Variability of the Terrestrial Hydrogen Exosphere

The increased geocoronal emissions during geomagnetic storms (Bailey and Gruntman 2013; Zoennchen et al. 2017) revealed the storm-time enhancement of exospheric hydrogen density. Cucho-Padin and Waldrop (2019) and Cucho-Padin et al. (2022) extracted time-dependent exospheric density variations from the TWINS geocoronal observations during a geomagnetic storm on 12-19 June 2008, by utilizing tomography. These studies suggested a dynamic response of the exospheric density in 3 – 8 $R_{E}$ to a geomagnetic storm. This motivated the MWG team to develop a physics-based exosphere model to understand the storm-time exospheric variability and its influence on the SMILE soft X-ray observations.

Connor et al. (2024) introduced a Model for Analyzing Terrestrial Exosphere (MATE). MATE follows similar modeling techniques used in a dynamic model of cusp ion dispersion (Connor et al. 2012, 2015). It has two main components: 1) a neutral hydrogen tracer and 2) an exospheric density calculator. The hydrogen tracer launches numerous neutral hydrogen atoms with various velocities at each exosphere grid and tracks them backward in time until the hydrogen atoms reach 500 km altitude (exobase-origin hydrogens), 100 $R_{E}$ radial distance (interplanetary hydrogens), or until their trace time in the exosphere reaches 50 days (satellite hydrogens). The tracer solves equations of motion in Geocentric Solar Ecliptic (GSE) coordinates using gravitational force with the Runge–Kutta 4th order method. The exospheric density calculator estimates phase-space densities (PSDs) of exobase-origin hydrogens assuming the neutral hydrogen atoms at 500 km altitude follow Maxwellian velocity distribution. Global circulation models like TIME-GCM and WACCM-X provide physics-based upper atmospheric conditions — hydrogen number density, neutral wind velocity, and temperature at 500 km altitude — the parameters for calculating the Maxwellian distribution. Then, the density calculator maps the PSDs back to the exosphere assuming Liouville’s theorem (i.e., conservation of PSDs along the particle trajectory) and obtains exospheric number density by integrating PSDs across velocity space.

The current version of the MATE simulation considers only gravity. It neglects the density contribution of interplanetary and satellite hydrogens, as well as source/loss processes along the hydrogen trajectory between the 500 km altitude and the exosphere. However, it utilizes computationally efficient backward tracing techniques, as well as the physics-based, storm-time upper atmosphere conditions from state-of-the-art global circulation models. These unique advantages allow MATE to study storm-time exospheric variability for the first time without the computational burden that the Monte Carlo model (or forward tracing model) often faces, especially for simulating the far-outside exosphere above 8 $R_{E}$, which limited previous modeling studies on a constant exosphere (e.g., Beth et al. 2016a, 2016b; Baliukin et al. 2019).

However, it is fair to say that Monte Carlo simulations consider more physical processes than MATE, such as solar radiation pressure, photoionization, neutral-neutral collisions, and neutral-plasma charge exchange, providing a deep understanding of each physical process’s impact on the time-independent exosphere. To address these missing physics, a series of MATE model improvements have been planned and are currently underway.

**Fig. 10:**
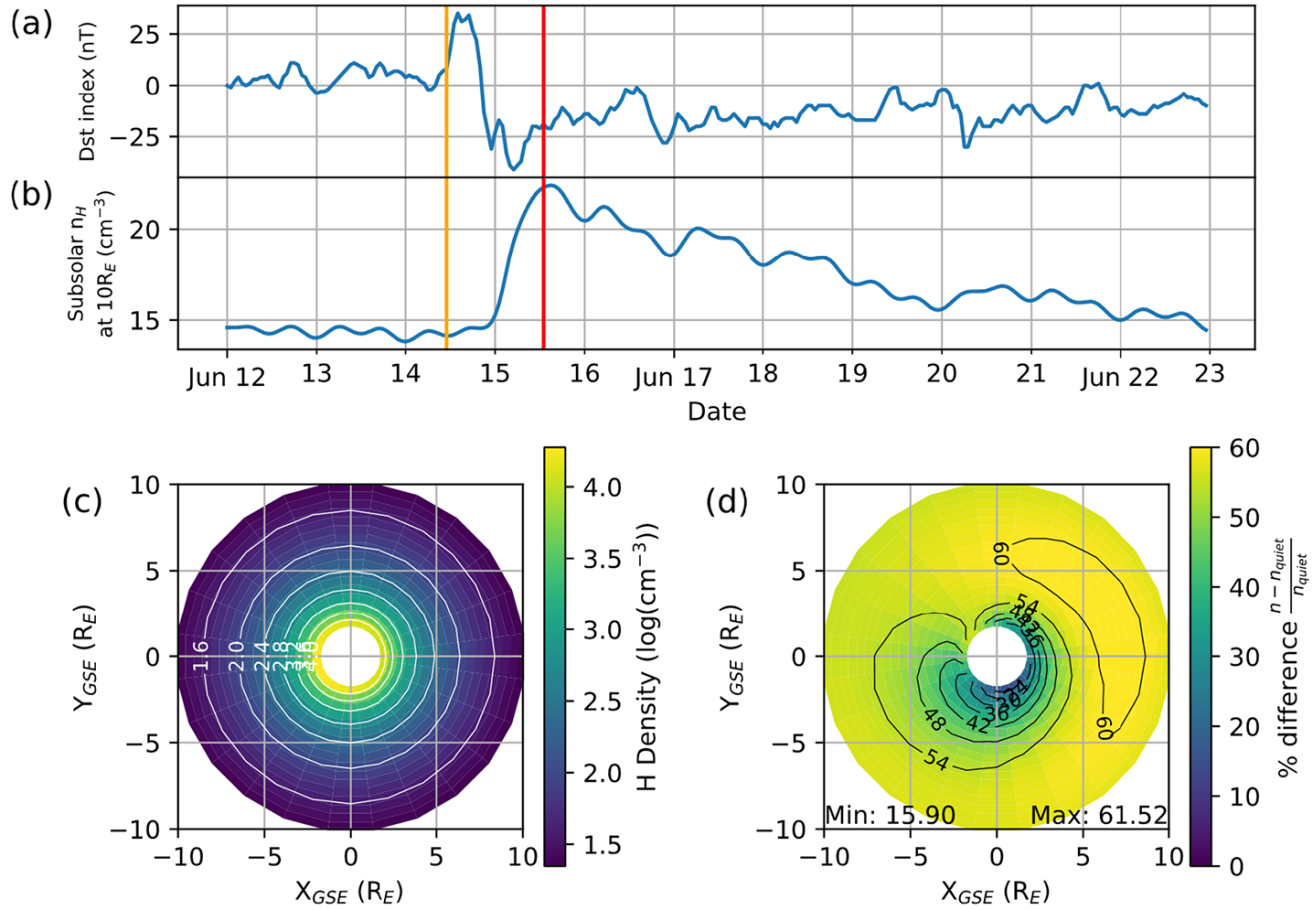
The MATE simulation results during 12 – 23 June 2008. (a) Dst index, (b) a subsolar exospheric hydrogen density at 10 $R_{E}$. (c) Storm-time exospheric neutral density on an ecliptic equatorial plane at 13 UT on 15 June 2008 (red vertical line in Fig. 10a-b), (d) percent increase of storm-time density with respect to quiet time at 11 UT on 14 June 2008 (orange vertical line in Fig. 10a-b). [Adapted from Connor et al. 2024]

Figure 10 presents the MATE simulation results during 12 – 23 June 2008. The top two panels show the Dst index and the subsolar exospheric hydrogen density at 10 $R_{E}$ ($N_{0}$), while the bottom panel displays the exospheric hydrogen density at an ecliptic equatorial plane. The left plot shows the storm-time exospheric density at 13 UT on 15 June 2008 while the right plot shows the percent difference of the storm-time density with respect to the quiet-time density at 11 UT on 14 June 2008. The red and orange lines in the top two panels indicate the storm and quiet times used in the bottom panel. The exospheric neutral density enhances globally but at a different rate, ∼30% near 2 $R_{E}$ and ∼62% near 7 $R_{E}$. The $N_{0}$ density also increases up to 64% from 14 to 23 cm^−3^ and remains elevated for more than 7 days. Connor et al. (2024) concluded that strong magnetospheric energy poured into the high-latitude ionosphere, initiating global thermospheric heating and thus providing more energy to neutral hydrogen atoms in the upper atmosphere entering the exosphere.

The MATE simulation shows ∼14 cm^−3^ at a 10 $R_{E}$ subsolar point during a quiet time, which is lower than 25 cm^−3^, the value recommended by MWG for soft X-ray simulations. As previously discussed, MATE considers only the gravitational force. Baliukin et al. (2019) reported that by including solar radiation pressure on top of the gravitational force, $N_{0}$ increases by ∼166%, from ∼15 to ∼40 cm^−3^. Based on their study, future model developments in MATE are scheduled to include solar radiation pressure, which is expected to increase $N_{0}$.

The MATE results in Fig. 10 provide interesting insights for the SMILE soft X-ray observations. During the initial phase of a geomagnetic storm, strong solar wind flux is expected, providing more soft-X ray source ions and thus enhancing the soft X-ray signal strength in the dayside magnetosheath. During the middle and end phases of the storm, solar wind flux may decrease to its typical value, but exospheric density may remain elevated for a longer period, thereby continuing to enhance the soft X-ray emissions. SMILE SXI may observe an entire storm due to the combined effect of elevated solar wind flux at the beginning of the storm and then the increased exospheric density throughout the storm duration.

## SMILE UltraViolet Imager (UVI)

The SMILE ultraviolet imager (UVI) is a CCD camera designed for imaging the Earth’s northern auroral region over the ∼160-180 nm UV waveband with a 10° × 10° field of view. UVI will serve to monitor the location and morphological variation of the northern auroral oval. Furthermore, the observed auroral intensities can also be used to infer certain key geophysical parameters such as the total energy flux of precipitating electrons. To assist in the design and performance evaluation of SMILE UVI, as well as to help achieve the scientific objective of UVI, MWG members at the University of Calgary and the University of Alberta in Canada have undertaken efforts in the following areas: (a) the development of an end-to-end simulator of UVI performance, and (b) the development of a synthesized electron transport model to simulate the kinetic transport of suprathermal electrons in the Earth’s atmosphere and their resulting UV emissions.

### Modeling the UVI Performance

The auroral imaging group at the University of Calgary, Canada, has developed an end-to-end simulator to evaluate the UVI performance. The inputs to the simulator include both the desired signals (auroras) and unwanted light sources (dayglow and scattered solar irradiance). The throughputs and outputs comprehensively consider the prototype design of the optics module (containing four mirrors and an MgF_2_ window) and detector module of SMILE UVI.

The University of Calgary has been developing an Auroral Transport Model (ATM) to support other optical imaging missions for several years (Liang et al. 2016, 2017, 2021, 2023). The ATM is designed to simulate the transport of suprathermal electrons in the upper atmosphere, including impact ionization and secondary/tertiary electron production, as well as the impact excitation of neutrals. The ATM enables the calculation of auroral and dayglow intensities in most pronounced emission lines/bands. Plasma density and temperature in the ionosphere can also be self-consistently computed. Depending on the research objective, the electron transport can be solved via either a fast simplified two-stream approximation (Solomon et al. 1988) or a more accurate yet time-consuming kinetic approach (see Sect. 5.2). The model will continue to be refined and is intended to serve as the support model (SMILE-ATM) for SMILE UVI.

To assess the influence of solar light backscattered into the view of satellite, the University of Calgary has developed another model to simulate the scattering of solar irradiance in the Earth’s atmosphere. Both Rayleigh scattering by atmospheric molecules and Mie scattering by aerosols are considered. The model was developed from the Simple Model for Atmospheric Radiative Transfer (SMART, Seidel et al. 2010), with standard atmospheric/aerosol profiles and a few key procedures (such as the multiple scattering routine) extracted from the Second Simulation of the Satellite Signal in the Solar Spectrum (6S) model (Vermote et al. 1997).

In the following demonstrative run of the simulator, a virtual satellite is positioned at 15 $R_{E}$ over the pole. The auroral oval is modeled according to the Holzworth and Meng (1975) parametrization of the Feldstein oval (Kp = 6), with a uniform total precipitation energy flux of 10 erg/cm^2^/s and a mean energy of 5 keV. The dayglow component is calculated using the solar EUV flux model described in Strickland et al. (1999), with the solar zenith angle geometry on 29 August 2019. The same date is also assumed in the solar irradiance scattering modeling. Full optical spectra of these light sources are modeled. The input signals reaching the front aperture of the satellite imager are processed as a function of the wavelength and the angle of incidence according to the viewing geometry. The incident signals are bandpass-filtered and converted into representative images via optics module modeling and detector module modeling. Image degradation according to the point spread function of the mirrors, the spectral responsivity of the detector, and representative noises such as the CCD readout noise, are all properly considered in the simulator.

**Fig. 11:**
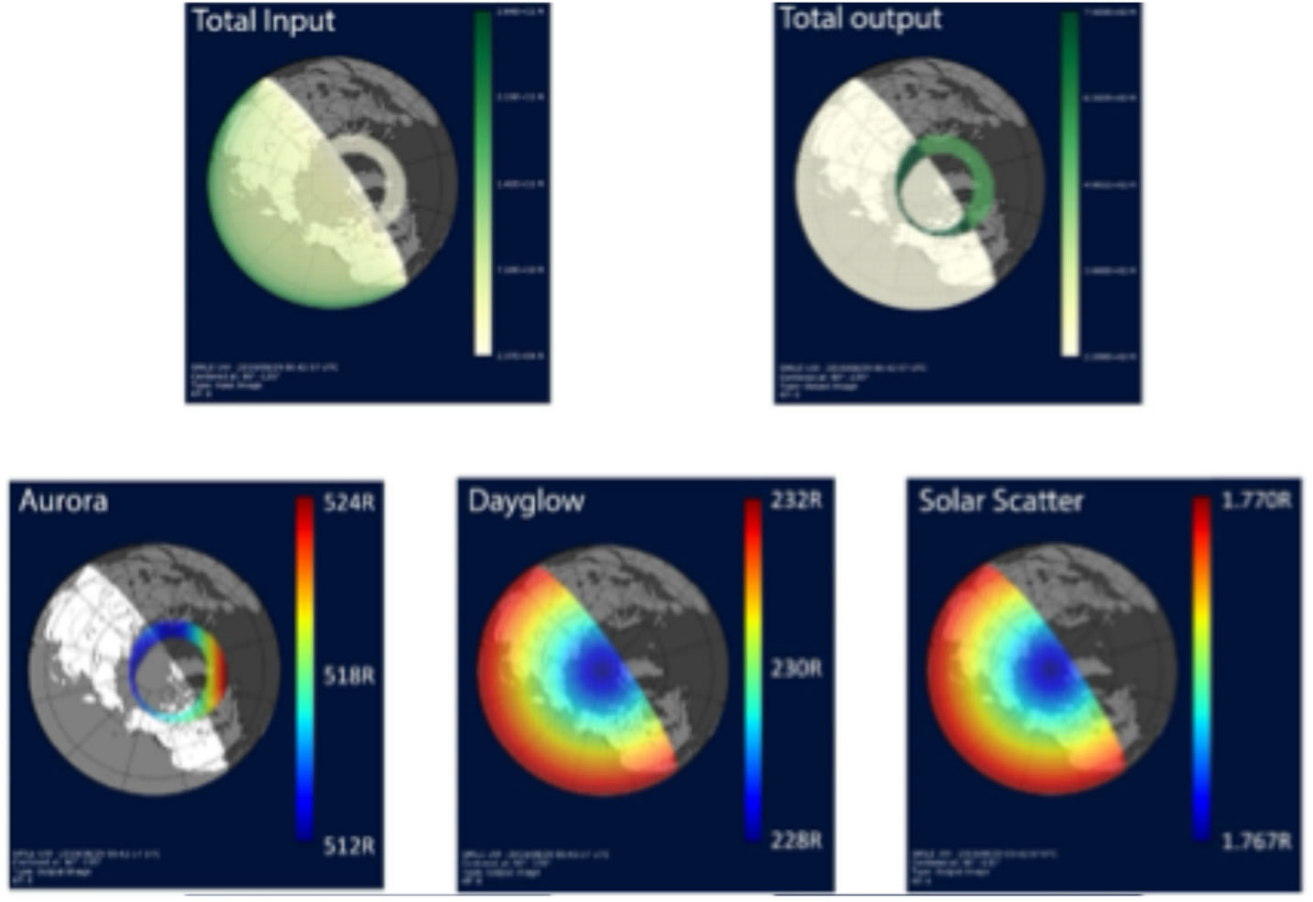
Top two panels show a stereographic view of the Earth with raw input lights (left-side panel) and the simulated output UVI image (right-side panel). Different shading indicates the sunlit/dark side. The bottom three panels display the individual components (aurora/dayglow/solar scattering) of the output image. Note that the color scale is different for each panel

The top two panels of Fig. 11 present a stereographic view of the Earth with raw input lights (left-side panels) and the simulated output UVI images (right-side panel). In the bottom three panels, we plot separately the individual components (aurora/dayglow/solar scattering) of the output image. The figure shows that the scattered solar radiance can be almost entirely out-of-band and has negligible influence on auroral observations for the planned SMILE UVI filter band. However, dayglow may constitute a non-trivial source of contamination on the dayside. Appropriate procedures to distinguish and subtract the dayglow component are thus necessary for UVI to achieve its scientific objective. Although the simulation was conducted in 2019-2020 based on a preliminary filter passband and prototype optics/detector module design, we have also tested with the final SMILE UVI filter specifications (Zhang et al. 2025) and confirmed that none of the main conclusions are qualitatively changed.

### Kinetic Auroral and Photo-Electron Transport and Resulting UV Emissions

To support SMILE UVI, the University of Alberta and the University of Calgary have jointly developed a model that synthesizes the kinetic transport of suprathermal electrons in conjugate ionospheres in the inner magnetosphere, and the electron impact excitation of UV emissions as well as their radiative transfer in the atmosphere. The model may serve two purposes. First, the model can be employed to evaluate the dayglow component which, as illustrated in Sect. 5.1, may constitute a nontrivial contamination source for the dayside auroral observation. Second, the model may pave the way to the inversion of precipitation parameters, such as the total precipitation energy flux, from UVI data.

The kinetic electron transport model simulates the transport of suprathermal electrons along a geomagnetic field line. These suprathermal electrons are described as gyro-particles. Primary source electrons can be either auroral electrons or photoelectrons produced by solar EUV photons. Secondary/tertiary electrons are generated via collisional ionization of atmospheric neutrals by the primary electrons. For the solar EUV flux spectrum input, the model can adopt either an empirical model (e.g., Strickland et al. 1999) or a realistic solar spectrum measured by spacecraft such as TIMED (Woods et al. 2000). Common empirical models such as NRLMSISE, IRI, and GCPM (Picone et al. 2002; Bilitza et al. 2022. Gallagher et al. 2000) are employed to obtain ambient neutral and plasma densities. Collisions between suprathermal electrons and neutrals are described using standard methods applied in kinetic particle-in-cell simulations (Vahedi and Surendra 1995). In total, the model accounts for 52 kinds of electron-neutral collisions with cross-sections described in Itikawa and Ichimura (1990), Majeed and Strickland (1997), and Itikawa (2006, 2009). Energy loss due to collision with thermal electrons is considered (Swartz et al. 1971). Coulomb scattering of electrons is also taken into account and described using the model of Nanbu (1997). Between successive collisions, photoelectrons are advanced along the field line under the conservation laws of energy and magnetic moment, with reflection at mirror points considered. Ultimately, the model yields the steady-state distribution of photoelectrons over energy and pitch angle along the field line.

Based on the suprathermal electron fluxes obtained with the model described above, we can then calculate the excitation rates of UV emissions and their radiative transfer in the Earth’s atmosphere. The chemistry and optical emission module is inherited from the previous Transition Region Explorer Auroral Transport Model (TREx-ATM) (Liang et al. 2016, 2017, 2021, 2023). To support the SMILE UVI, the model is now capable of simulating the most prominent lines/bands of the UV emissions in the ∼100-250 nm wavelength range, including the N_2_ Lyman-Birge-Hopfield (LBH) band system, the OI 130.4/135.6 nm, and the NI 149.3 nm, etc. These UV emissions undergo radiative transfer, including scattering and absorption in the atmosphere (Meier 1991). Such radiative transfer of UV emissions is modeled with appropriate methods contingent on the nature of specific UV lines/bands, e.g., O_2_ pure absorption for LBH, and O resonant scattering for 130.4/135.6 nm.

The synthesized model simulates photoelectron production and interhemispheric transport, as well as the resulting UV emissions. In particular, the model was applied to a phenomenon known as “anomalous emission” (Kil et al. 2020), which was hypothesized to be caused by interhemispherically transported photoelectrons (IHTPE) from the sunlit hemisphere to the dark hemisphere.

**Fig. 12:**
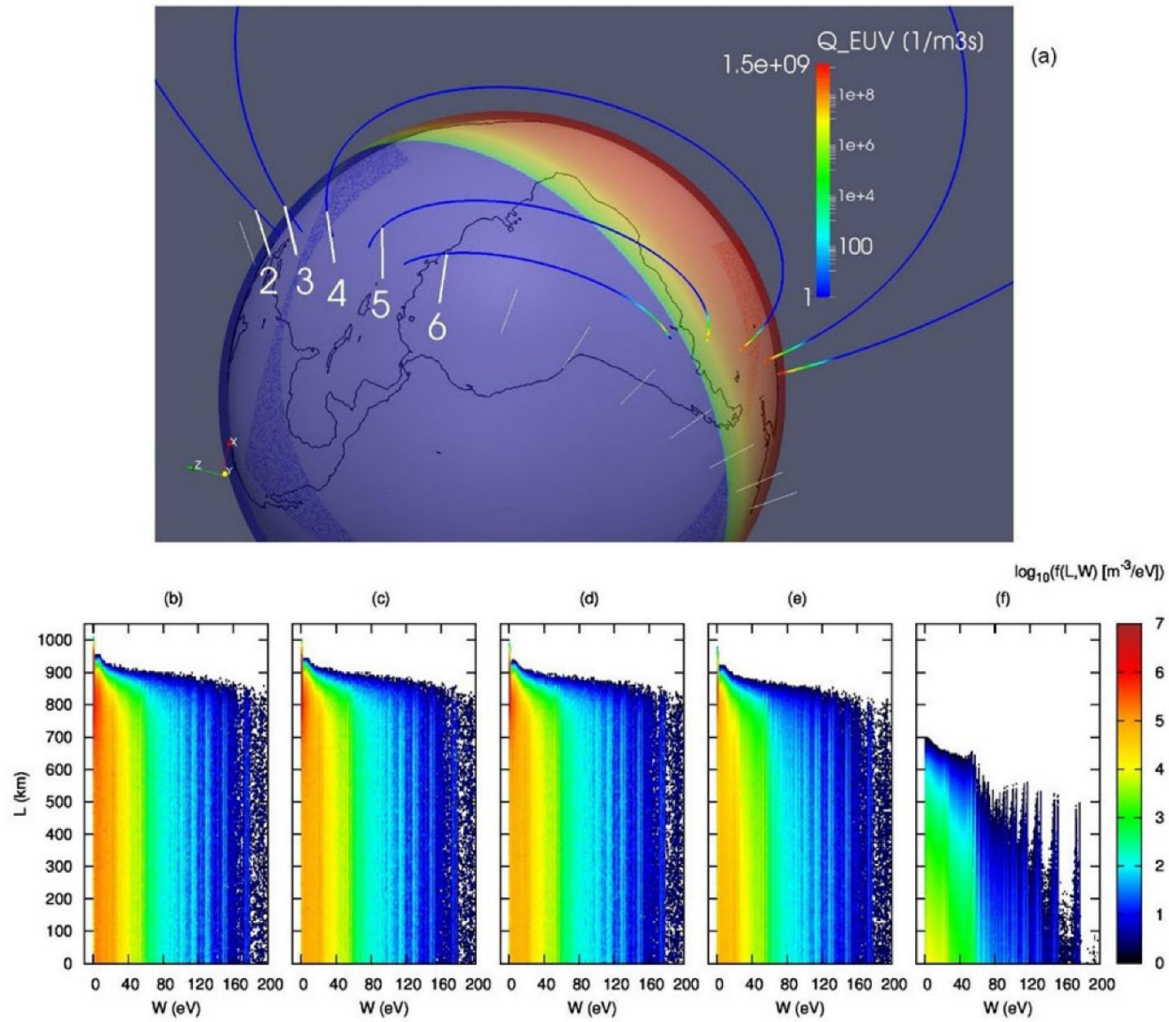
(a) General view of the system where propagation of photoelectrons was calculated. The white straight lines are the lines of sights for the 13 orbital points used in the study. Thick white straight lines with labels 2 to 6 represent orbital points the energy spectra of which are shown in panels (b-f), respectively. The blue curves are the dipole geomagnetic field lines passing through the spacecraft positions (top ends of the lines of sight). The colored transparent sphere shows total ionization rates by the solar EUV at altitude 200 km at UT 8.060773h (orbital point 3). The field lines are colored according to the value of the total ionization rate by the solar EUV using the same color scheme as the sphere. (b-f) Density of photoelectrons vs photoelectron energy (the horizontal axis) and distance from the spacecraft along the line of sight (the vertical axis). The distance increases towards the Earth

Figure 12 shows an event example on 1 January 2017. 13 orbital points are sampled along the orbit of the DMSP F16 spacecraft, with UT uniformly distributed between 7.96h and 8.58h. A line-of-sight (LOS) is created according to the viewing direction of the Special Sensor Ultraviolet Spectrographic Imager (SSUSI, Paxton et al. 2002) onboard DMSP, for each of the 13 orbital points (see Fig. 12a). The solar EUV flux spectrum is obtained from the measurements by the TIMED/Solar EUV Experiment (Woods et al. 2000) on the event date. The kinetic electron transport model described above is applied to obtain the photoelectron flux distribution along the LOS. In Fig. 12, the orbital points 2-6 are numbered, and their corresponding photoelectron distributions along the LOS are plotted in Figs. 12(b-f), respectively. These points are in the northern hemisphere in the night sector, but geomagnetic field lines through the LOSs of these points have southern ends in the day sector, resulting in IHTPEs. Points 2-5 and their corresponding LOSs have their conjugate footprints in the southern hemisphere fully under sunlit. Photoelectron energy spectra along the LOS of points 2-5 are similar, with values gradually decreasing as their conjugate sunlit footprints increase in solar zenith angle. Field lines through the upper part of the LOS at orbital point 6 have segments in the southern hemisphere partially illuminated by the Sun, although the light rays go nearly parallel to the Earth’s surface which increases the optical depth. Thus, the photoelectron energy spectrum along the LOS of point 6 (Fig. 12f) is much weaker than that for points 2-5.

**Fig. 13:**
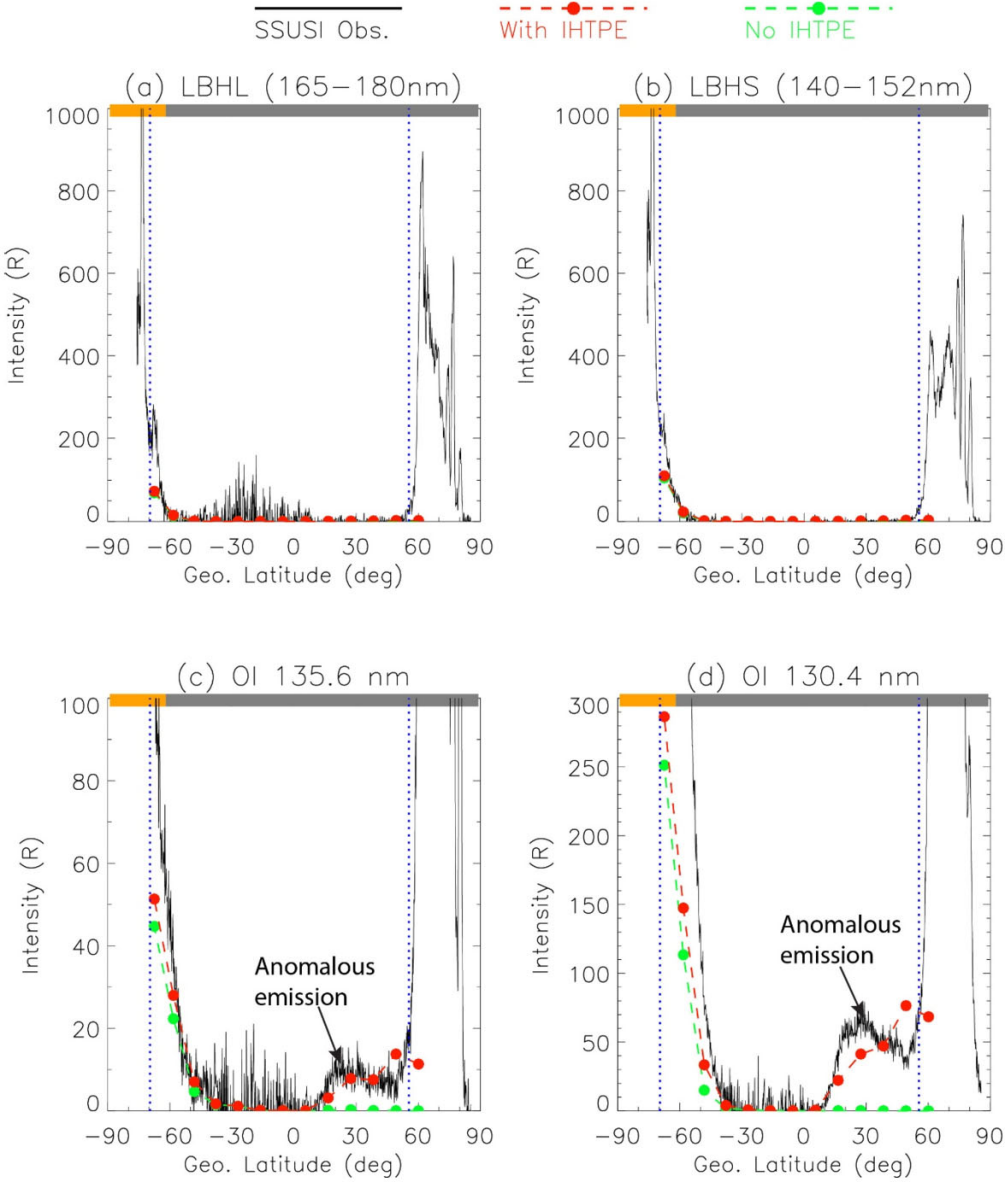
Comparison of model simulation and SSUSI observations for the 01-Jan-2017 event. A pierce-point altitude of 150 km is used in calculating the latitude for each orbital point. In all subfigures, a black curve denotes the SSUSI observations; a red line with dots denotes the modeled emission intensity with IHTPE, while a green line with dots denotes the modeled emission intensity without IHTPE. Two vertical dotted lines in each figure mark the equatorward boundary of electron auroral precipitation in two hemispheres inferred from the SSJ data. On top of each subfigure we mark the sunlit region (yellow bar) and the dark region (gray bar) for the passage

Figure 13 compares modeled emission intensities and those observed by SSUSI. Note that auroras at high latitudes in both hemispheres are not of interest here since this study only involves photoelectrons. The sunlit region is in the southern hemisphere, and the rise of modeled LBHL/LBHS emission intensities south of $\sim -60$° GLAT indicates the satellite transition into the sunlit region. Outside the sunlit region, dayglows diminish as expected. However, as shown in Fig. 13c and 13d, a region of moderate emissions exists between ∼10° and 50° GLAT. We follow Kil et al. (2020) to term them “anomalous emissions”. Such anomalous emissions are evidently manifested in OI 135.6 nm and 130.4 nm lines. The modeled OI emission intensities correctly predict the rise of anomalous emissions poleward of ∼10° GLAT, and reasonably agree with the realistic SSUSI observations.

To better demonstrate the influence of conjugate photoelectrons, we also calculate UV emission intensities without the interhemispheric electron transport. Local photoelectron fluxes in the sunlit hemisphere are kept intact in the calculation. Under these circumstances, the emission intensity is very small all over the dark region. The comparison among the modeled emission intensities with and without conjugate photoelectrons and realistic SSUSI observations, clearly indicates that the IHTPEs are the most likely source of the anomalous emission.

To summarize, our model results agree reasonably well with the DMSP/SSUSI observations and have reproduced the nightside anomalous emission led by the IHTPE. The modeled LBH intensities are also in reasonable agreement with the observed LBH dayglows. Scientifically, the results corroborate that nightside anomalous emission is caused by conjugate photoelectrons from the sunlit hemisphere. Technically, this study demonstrates our model’s ability to model UV emissions for the SMILE UVI mission.

In addition, there are a few ongoing modeling efforts that can potentially provide more support for SMILE UVI. These include a recently-developed proton transport model designed to simulate the proton auroras in response to energetic proton precipitation (Liang et al. 2024), and additional validation of the photoelectron model by comparison with the Atmospheric Explorer E measurements.

## Summary

The SMILE MWG has actively supported the SMILE mission by conducting simulations of SMILE soft X-ray images, developing various SXI/UVI analysis techniques, and promoting SMILE-related scientific research (e.g., storm-time exosphere variability and UV emissions caused by interhemispherically transported photoelectrons). The MWG also fostered team discussions through monthly virtual meetings and semi-annual in-person meetings to ensure the best outcomes for the SMIE mission. This paper covers only a subset of the MWG activities related to the SMILE SXI and UVI images. Interested readers are encouraged to follow a special issue on SMILE MWG activities (Sun et al. 2024 and references there in). Below is a summary of this paper.

Firstly, we introduced how to simulate SMILE SXI images of the Earth’s dayside magnetosphere system. Soft X-ray imaging is new to the heliophysics community, though well-known in astrophysics. MWG simulated expected soft X-ray images using a global magnetosphere model and a simple exospheric neutral density model, considering SMILE’s orbit, SXI look direction, and SXI field of view. The resultant images enhance our understanding of SXI performance. SXI can capture the global scale of solar wind – magnetosphere interactions, such as Earthward magnetopause motion during southward IMF turning and the enhancement of dynamic solar wind pressure. However, SXI has limitations in capturing transient localized phenomena like FTEs and high-speed magnetosheath jets unless such structures and their surroundings emit strong X-rays within the SXI field-of-view.

Secondly, we discussed various techniques for analyzing SXI images. We introduced a suite of techniques to identify magnetopause and cusp locations from 2D soft X-ray images, even under low signal-to-noise (SNR) conditions. Additionally, we presented several AI techniques for extracting meaningful information from synthetic SXI images, such as detecting angles of soft X-ray peaks, verifying magnetopause presence within SXI FOV, and enhancing noisy SXI images. By combining these techniques, we can extract the location and motion of magnetopause and cusps for a variety of solar wind conditions and SMILE vantage points.

Thirdly, we introduced several studies on exospheric neutral density, a key parameter determining near-Earth soft X-ray emissions. MWG has used 25 cm^−3^ as the exospheric neutral density at a 10$R_{E}$ subsolar location ($N_{0}$) for soft X-ray calculations. Recent studies on geocoronal and soft X-ray observations suggest that 25 cm^−3^ is a conservative yet appropriate value, validating MWG’s choice of this density. Additionally, MWG developed a new Model for Analyzing Terrestrial Exosphere (MATE), revealing that $N_{0}$ can increases by up to 64% shortly after Dst hits its minimum and that this density remains elevated for up to 7 days. This suggested that SMILE would observe bright SXI images at the onset of a geomagnetic storm due to increased solar wind flux, and continuously throughout the storm due to elevated exospheric density.

Finally, we reviewed the UVI modeling efforts. The UVI simulator generates synthetic UV images of the northern hemisphere, incorporating desired signals such as auroras and unwanted light sources like dayglow and scattered solar irradiance. This simulator also considers the protype design of the optics and detector modules of SMILE UVI. Their findings suggest that scattered solar irradiance minimally affects auroral observations, while dayglow may introduce non-trivial contamination in dayside auroral observations. Additionally, a kinetic electron transport model is developed and applied in a study of the transport of photoelectrons from the sunlit hemisphere to the dark hemisphere and their impact on UV images. Their model results show good agreement with the DMSP/SSUSI UV observations and suggest that the nightside UV emissions are primarily due to photoelectrons originating from the conjugate sunlit hemisphere. This demonstrates the applicability of the kinetic electron transport model in simulating UV emissions for SMILE UVI.
